# Gaze-contingent reinforcement learning reveals incentive value of social signals in young children and adults

**DOI:** 10.1098/rspb.2016.2747

**Published:** 2017-03-01

**Authors:** Angélina Vernetti, Tim J. Smith, Atsushi Senju

**Affiliations:** Centre for Brain and Cognitive Development, Department of Psychological Sciences, Birkbeck, University of London, Malet Street, London WC1E 7HX, UK

**Keywords:** social attention, orienting, reinforcement learning, eye-tracking, gaze-contingency, development

## Abstract

While numerous studies have demonstrated that infants and adults preferentially orient to social stimuli, it remains unclear as to what drives such preferential orienting. It has been suggested that the learned association between social cues and subsequent reward delivery might shape such social orienting. Using a novel, spontaneous indication of reinforcement learning (with the use of a gaze contingent reward-learning task), we investigated whether children and adults' orienting towards social and non-social visual cues can be elicited by the association between participants' visual attention and a rewarding outcome. Critically, we assessed whether the engaging nature of the social cues influences the process of reinforcement learning. Both children and adults learned to orient more often to the visual cues associated with reward delivery, demonstrating that cue–reward association reinforced visual orienting. More importantly, when the reward-predictive cue was social and engaging, both children and adults learned the cue–reward association faster and more efficiently than when the reward-predictive cue was social but non-engaging. These new findings indicate that social engaging cues have a positive incentive value. This could possibly be because they usually coincide with positive outcomes in real life, which could partly drive the development of social orienting.

## 1. Introduction

A significant amount of research has investigated humans' preferential attention towards social signals such as facial features, emotion, gaze direction, head orientation and speech [1–4], as such signals are fundamental to day-to-day social communications and social learning. An important question about social attention is how humans develop a preference for, and attentional orienting to, socially relevant signals. Three non-mutually exclusive theories have proposed different mechanisms underlying social orienting. Firstly, social stimuli may attract automatic attention, possibly due to a human predisposition present from birth, that guide an infant's orienting towards socially relevant signals such as faces [5,6]. Secondly, social stimuli may possess an intrinsic hedonic value, which generates pleasure (i.e. primary reinforcement) and motivates social orienting [7,8]. Thirdly, social stimuli may acquire incentive value through associative learning (i.e. secondary reinforcement) [9]. In other words, humans might preferentially orient to social signals because they usually predict subsequent reward deliveries in daily life [10].

Such associative learning has been shown to influence visual attention in the non-social domain. In early development, infants orient faster and look longer towards a colourful shape that is predictive of the delivery of a reward (i.e. animated cartoon or pictures) [11,12]. Similarly, several studies showed that non-social visual stimuli (i.e. shapes), associated with a positive outcome (i.e. monetary or pictures), influence attentional selection in adolescents and adults [12–17]. This demonstrates that, when associated with a positive outcome, non-social visual stimuli influence selective orienting throughout the lifespan.

Associative learning has also been shown to influence visual attention in the social domain. Infants' attention has been shown to be modulated by the learned association between non-social stimuli and the appearance of an audio-visual outcome, when the stimuli are repeatedly preceded by communicative signals such as an adult addressing infants [18]. In a recent study, non-social stimuli (i.e. shape), previously associated with a positive social feedback elicited attentional capture in adults [19], demonstrating that the association between the display of stimuli and accompanying social signals can also modulate adults' gaze behaviour. By contrast, other studies suggested that social signals, unlike primary reinforcers, do not always trigger preferential orienting. Infants have been shown to preferably follow others' gaze, and to look longer to a face gazing at an object compared with a face gazing away from an object, only when the gaze shift was preceded by a period of eye contact or infant directed speech [20,21]. This suggests that preferential orienting depends on infants' preference for informative social signals. Infants also orient faster towards a stimulus that is non-social but predictive of the delivery of a reward [11], despite the presence of a visual social distractor, such as a smiling face. Similarly, adults have been shown to attend less to the eye region compared to the mouth region when watching someone talk [22] suggesting that attending to social signals is context-dependent.

The evidence presented above seems to be consistent with the hypothesis that the incentive value of social stimuli, or the experienced association between social signals and subsequent reward delivery, can account for the preferential orienting towards social stimuli, or at least part of it. However, no prior study has directly tested this hypothesis by measuring the spontaneous gaze of the participants. Do adults and children preferentially attend to social signals because they are more likely to predict positive outcomes? If so, do social signals displaying more engaging features, such as positive affect or referential action, attract attention because they are more likely to be associated with positive outcomes in real life?

To address these questions, the current study investigated whether a reward-predictive cue modulates spontaneous visual orienting differently depending on whether it has social and engaging characteristics compared with non-social and/or non-engaging features. To achieve this aim, a gaze-contingent learning paradigm was devised, in which the visual fixation on one of two alternative cues triggers the delivery of a reward, and fixation on the other alternative triggers the delivery of a penalty. In order to evaluate the influence of the social versus non-social signals, and engaging versus non-engaging signals, those cues were associated with the reward or penalty in different conditions. Based on previous research looking at different social engaging cues, such as happy versus negative facial expressions [23], and referential versus non-referential head turns [24], the engaging stimuli in our study were defined as positive audio-visual signals with a reorienting of the stimuli toward the location of subsequent reward delivery. By contrast, the non-engaging stimuli were defined as negative audio-visual signals with a reorienting of the stimuli away from the location of subsequent reward delivery. Concretely, the engaging social cues included positive emotional facial expression and voice, and referential head turn, i.e. head orientation cueing towards the location of the reward. The non-engaging social cues consisted of negative emotional facial expression and voice, and non-referential head turn, i.e. head orientation cueing away from the location of the reward. We additionally created corresponding non-social cues (engaging and non-engaging), which consisted of videos of two dynamic spheres containing an arrow. These stimuli closely matched the respective social cues in terms of spatio-temporal dynamics as well as affective and referential contents, in order to control for any effect of low-level features. The engaging non-social cues consisted of lighter colour change and positive non-social sound, followed by referential turn of an arrowhead towards the reward. The non-engaging non-social cue consisted of darker colour change and negative non-social sound, followed by non-referential turn of an arrowhead away from the reward.

We made three predictions. Firstly, based on previous research, we predicted that both children and adults would attend more frequently to the cues that trigger the reward delivery. Secondly, compared with social but non-engaging cues, we predicted that socially engaging cues would influence cue–reward learning, either because (i) social engaging cues habitually predict the subsequent delivery of a positive outcome in real-life, or (ii) because engaging social cues are preferably selected in the environment due to their intrinsic hedonic value or due to automatic orienting. These two alternatives can be dissociated in the condition in which socially engaging cues do not trigger the reward delivery, with the former predicting slower cue–reward learning and the latter predicting preference towards engaging cues over non-engaging cues. Finally, we predicted that adults, like children, would be influenced by socially engaging cues but could achieve the task more efficiently and/or more rapidly than children, as they may be more experienced with cue–reward associations.

## 2. Methods

### (a) Participants

Four groups of 16 children (35 females, *M*: 3.31 years, s.d.: 0.24) and four groups of 16 adults (43 females, *M*: 28.20 years, s.d.: 7.71) completed the study. An additional 11 children and 10 adults were excluded from the analyses because of (i) refusal to remain seated facing the monitor (one child), (ii) refusal to face forward in the high-chair (five children), (iii) staring in the middle of the screen for more than 5 min (two adults) or (iv) falling asleep in front of the screen (two adults), (v) poor eye tracker calibration (two children, three adults) and (vi) equipment failure (three children, three adults). The exclusion rate for children is within the typical range of eye-tracking studies with young children, as reported in a meta-analysis in infant study [25]. The exclusion rate for adults is similar to the adult dropout rate reported in a study using a comparable gaze-contingent paradigm [12]. Children were recruited by advertising in parent friendly magazines. They were offered a t-shirt or a colouring book for their participation, and their parents were reimbursed for their travel expenses. Adults were recruited via the University's experiment management system and were remunerated with credits or a financial compensation of £8. The procedure was approved by the Research Ethics Committee of the Department of Psychological Sciences, Birkbeck, University of London.

### (b) Apparatus and task

**Figure 1.**
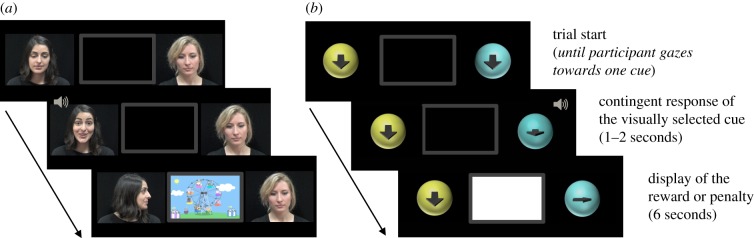
Sequence of events in a single trial. A trial starts with the first frame of two cues displayed on each side of a screen (two faces looking downward in the social condition (*a*), and two discs with a downward-pointing arrow in the non-social condition (*b*)). Looking at one of the two cues for a minimum of 400 ms triggers the display of the corresponding cueing video sequence. In the social condition, when fixated, an engaging person greets and turns towards the frame while the other non-engaging person moans and turns away from the frame. In the non-social condition, an engaging disc displays an arrow associated with a winning jingle ding and the arrow then points towards the frame while the other non-engaging disc displays an arrow associated with a failing jingle dong and the arrow then points away from the frame (*b*). Depending on whether the engaging or non-engaging cues are reward predictive or not, a reward (animated cartoon) or a penalty (blank screen) is then displayed in the middle of the screen for 6 s (see electronic supplementary material, video S1 for cueing sequences). (Online version in colour.)

The gaze contingent task was created using MatLab [26], the Psychophysics Toolbox extensions [27–29] and Tobii Analytics Software Development Kit [30]. The participants' gaze was recorded during the task via an eye tracker Tobii TX300 (120 Hz sampling rate, 23-inch monitor, situated 60 cm away from the participants). The task consisted of the display of two dynamic cues (width/height: 11.5°/8.7°) presented on each side of a picture representing the frame of a television, and depending on the participant's response, either a reward or a penalty (width/height: 21.3°/11.8°) presented in the centre of the frame (figure 1). Each trial started with the presentation of both the reward-predictive and the non-reward-predictive cues in a non-responsive initial display. The participants' fixation on one of the two cues for a minimum of 400 ms triggered the display of the corresponding video sequence of the cue, which was followed by either the display of the reward (popular animated cartoon) or the penalty (blank screen) for 6 s. At the end of the reward or penalty delivery, two cues resumed their initial states (e.g. faces/arrows pointing down), seamlessly starting the next trial. The cues were displayed for an unlimited amount of time until a participant gazed at one of the two cues. On average, it took 1.2 s for the participants to trigger one of the two cues. The task consisted of a total of 40 trials. Two conditions were implemented to investigate the influence of the social nature of the cues (social or non-social cues). The engaging nature of the reward-predictive cues (engaging or non-engaging cues) was investigated in each social and non-social condition across participants.

### (c) Stimuli

**Figure 2.**
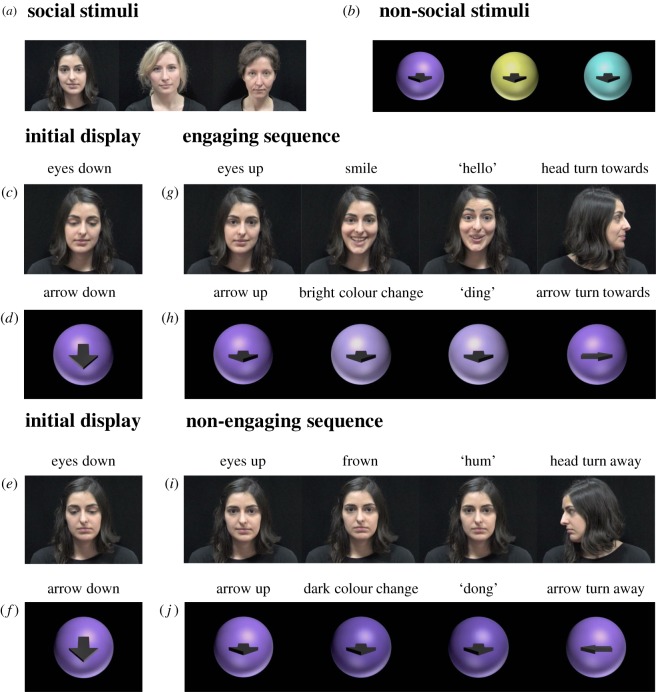
Stimuli used in both social and non-social conditions and for both engaging and non-engaging cues. Three different faces (*a*) and three different spheres (*b*) were employed in pairs and counterbalanced across participants. A trial started with the initial display of the cues remaining still until the participants' gaze (*c*–*f*). When gazed at, the engaging or non-engaging sequence of each cue would be displayed for both social (*g*) and (*i*) and non-social conditions (*h*) and (*j*). (Online version in colour.)

Four different types of dynamic cues (social engaging, social non-engaging, non-social engaging and non-social non-engaging) were created (figure 2 and electronic supplementary material, video S1). The social cues consisted of female actors with face and shoulders visible (figure 2*a*). The non-social cues consisted of spheres containing an arrow (figure 2*b*). Since both reward-predictive and non-reward-predictive cues were non-responsive at the beginning of a trial (figure 2*c*–*f*), the initial display of the cues was characterized by different features to allow differentiation and recognition: in the social condition, two different identities were used (but with similar neutral expression) and in the non-social condition, the two spheres had two different colours (but otherwise similar features). We also attempted to minimize the potential animacy or agency cues portrayed by the non-social stimuli, because these cues could inadvertently elicit perception of social agency and generate social cognition such as gaze following [31,32]. The video sequences of the cues consisted of three comparable main phases. The first phase corresponded to an initiation of response to the participants' gaze. More specifically, when the participant gazed at one of the cues, the cue initiated a response: the females' eyes (in the social condition) or the arrow inside the spheres (in the non-social condition) moved toward the direction of the participant (i.e. directly out of the screen). The second and third phases corresponded to a responsive audio-visual display, as well as a cueing movement, which differentiate between engaging and non-engaging stimuli. More specifically, in the engaging stimuli, the social cue consisted of a person displaying a smile associated with the positive exclamation ‘hello’ and the head turning towards the location of the subsequent reward delivery (figure 2*g*). Similarly, the non-social cue consisted of a sphere displaying a bright colour change associated with the sound ‘ding’ and the arrow turning towards the location of the subsequent reward delivery (figure 2*h*). In the non-engaging stimuli, the social cue consisted of a person displaying a frowning expression associated with the negative exclamation ‘hum’ and the head turning away from the location of the subsequent reward delivery (figure 2*i*). Similarly, the non-social cue consisted of a sphere displaying a dark colour change associated with the sound ‘dong’ and the arrow turning away from the location of the subsequent reward delivery (figure 2*j*; electronic supplementary material, video S1).

The engaging and non-engaging stimuli used in the social and non-social conditions were carefully designed to be as spatially and temporally matched as possible in their audio-visual dynamics, affective signal (second phase) and referential signal (third phase). The vocal and non-vocal sounds used in the social and non-social conditions include features that are commonly characterized to convey positive and negative signals. For the social condition, two commonly used exclamations (‘hello’ and ‘hum’) have been selected to contrast positive and negative utterances. For the non-social condition, two non-social sounds ‘ding’ and ‘dong’ were used to convey positive and negative signals. To further validate the stimuli, we asked 15 adults (age, *M* = 23.85 years, s.d. = 5.53 years, 11 females), who were naive to the purpose of the study and different from the participants taking part in the main experiment, to rate the two non-social sounds on a nine-point scale from very negative (−4) to very positive (4), as well as from very non-engaging (−4) to very engaging (4). The ratings were converted from −4 to 4 to an ordinal scale of 1–9. Wilcoxon signed-rank tests confirmed that the sound ‘ding’ was rated significantly more positively (*M* = 6.47, s.d. = 1.92) than the sound ‘dong’ (*M* = 3.40, s.d. = 2.29, *Z* = 2.31, *p* = 0.021). Similarly, the sound ‘ding’ was rated significantly more engaging (*M* = 6.67, s.d. = 2.19) than the sound ‘dong’ (*M* = 4.00, s.d. = 1.96, *Z* = 2.85, *p* = 0.005).

### (d) Design and procedure

A full between-subject design was employed, to make sure that each participant experienced only one combination of a cue and a reward. This design was selected to avoid a possible carry-over effect. Indeed, a learned association between a certain type of cue and reward delivery in a previous task might generate inadvertent value attribution to such a cue, which could affect learning in subsequent tasks. Participants started the task after a child-friendly gaze-contingent calibration that consisted in the presentation of bouncing animations at the four corners and the centre of the screen. Participants were instructed to watch and keep watching the screen, but no further instruction was provided. The cues were presented in the same location five times in a row to allow expectation to develop, but switched to the opposite side in every five trials to control for any potential spatial bias. Although the reaction of both engaging and non-engaging cues were only displayed if the participants gazed at the respective cues, the participants were exposed to the video sequences of both engaging and non-engaging cues from the very beginning of the task (see electronic supplementary material). In both social and non-social conditions, three different pairs of cues (figure 2*a*,*b*) and four different cartoons were used, which were counterbalanced across participants. To assess any preference for either cues at the beginning and at the end of the task, a pre, and post-test consisting in the display of the two cues were presented for 10 s. Following the end of the experiment, the participants were asked feedback questions investigating awareness of the gaze contingency and appreciation of the cartoon reward (see electronic supplementary material).

### (e) Measures

To assess reward learning, the participant's first gaze shifts (rewarding first looks) in which a fixation of at least 400 ms duration landed on the reward-predictive cue, were analysed in both social and non-social conditions, and for both types of rewarding cue (engaging and non-engaging). In order to assess learning over the course of the task, four 10-trial blocks, in which the reward-predictive cue was presented an equal number of times on each side of the screen, were selected. Additionally, the proportion of looking time towards the reward-predictive cue at pre-test and post-test was calculated for the first 5 s (looking time towards the reward-predictive cue divided by the total looking time towards both the reward-predictive and penalty-related cues).

## 3. Results

### (a) Chance level comparisons

**Figure 3.**
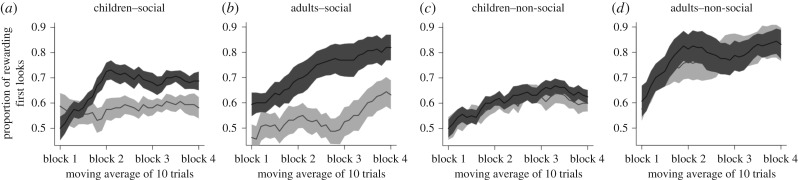
Moving average of the proportion of rewarding first looks (moving average of 10 consecutive trials) in both social (*a* and *b*) and non-social conditions (*c* and *d*) for both children (*a* and *c*) and adults (*b* and *d*). Statistical analyses were carried out using four independent 10-trial blocks (1–10, 11–20, 21–30 and 31–40 trials blocks, see the results section for details). Engaging reward predictive cue in dark grey. Non-engaging reward predictive cue in light grey. Solid black lines: average of the proportion of rewarding first looks of 10 consecutive trials; dark grey and light grey shaded areas: standard errors.

To investigate whether the performance of each group for each reward-predictive cue and block was different from chance level, the proportions of rewarding first looks were compared against a probability of 0.5. In the social condition with engaging cues, both groups of children and adults performed above chance level during the last three blocks (all *t*_15_ > 3.30, all *p* < 0.005, all *d* > 0.82, figure 3*a*,*b*, dark grey line). However, in the social condition with non-engaging cues, the group of children performed above chance level only during the third block (*t*_15_ = 2.78, *p* = 0.014, *d* = 0.69, figure 3*a*, light grey line) and a similar trend was observed during the last block (*t*_15_ = 1.98, *p* = 0.066, *d* = 0.49). The group of adults performed above chance level only during the last block (*t*_15_ = 2.34, *p* = 0.033, *d* = 0.58, figure 3*b*, light grey line). In the non-social condition with both engaging and non-engaging cues, both groups of children and adults performed above chance level during the last three blocks (all *t*_15_ > 3.16, all *p* < 0.006, all *d* > 0.79, figure 3*c*,*d*). In addition, the analyses of the proportion of looking time revealed that the group of adults looked significantly longer at the rewarding-predictive cue than the penalty-predictive cue at post-test but not at pre-test. This was not the case for the group of children, possibly because of a strong side bias, which could mask any preferential looking for the reward-predictive cue at post-test (electronic supplementary material, figures S1*a*–*d*). The details of this analysis as well as an additional side bias analysis during pre-test and post-test are reported in the electronic supplementary material.

### (b) Generalized estimating equation analysis

To examine the participants' performance over the course of the task, the first looks were entered in a generalized estimating equation analysis. The model was built with a binomial distribution, a logit link function, an unstructured correlation matrix and a robust estimator. For each trial, the participants' gaze shift towards the rewarding cue was coded as 1. If the participants gazed at the non-rewarding cue the trial was coded as 0. The factor block (blocks 1, 2, 3 and 4) was entered as within subject factor. The age (adults/children), the social nature of the cues (social/non-social) as well as the engaging nature of the cues (engaging/non-engaging) were entered as between-subject factors in the model. A follow-up model estimated the effects of the interactions among the factors.

The analyses revealed a significant main effect of block (Wald 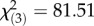, *p* < 0.001). The participants learned the cue–reward association and looked more frequently at the reward-predictive cue in the three last blocks compared with the first block (the proportion of choices towards the rewarding cues significantly increased over blocks, block 1 versus block 2, block 3 and block 4: all *p* < 0.001, block 2 versus block 3 and block 4: all *p* < 0.004 and block 3 versus block 4: *p* = 0.235).

A significant main effect of the engaging nature of the cues (Wald 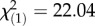, *p* < 0.001) as well as a significant two-way interaction between the social nature and engaging nature of the cues (Wald 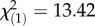, *p* < 0.001) were found. Follow-up contrasts revealed that the participants performed better with engaging reward-predictive cues than with non-engaging reward-predictive cues in the social condition (*p* < 0.001, figure 3*a*,*b*) but not in the non-social condition (*p* = 0.315, figure 3*c*,*d*). Participants made more rewarding choices when the cues were social and engaging compared to when the cues were non-social and engaging (*p* = 0.011). By contrast, they made less rewarding choices when the cues were social and non-engaging compared to when the cues were non-social and non-engaging (*p* = 0.005).

There was no overall effect of age (Wald 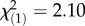, *p* = 0.147) but a significant two-way interaction between age and the engaging nature of the cues was found (Wald 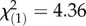, *p* = 0.037). Indeed, the group of adults made more rewarding choices when the reward-predictive cues were engaging than the group of children did (*p* = 0.007). By contrast, both groups of children and adults performed similarly when the reward-predictive cues were non-engaging (*p* = 0.876). There was no significant two-way interaction between age and the social nature of the cues or three-way interaction between age, the social nature of the cues and the engaging nature of the cues (all Wald 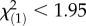, all *p* > 0.163).

In addition, the analyses revealed two significant two-way interactions between block and the engaging nature of the cues (Wald 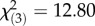, *p* = 0.005) and between age and block (Wald 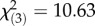, *p* = 0.014) as well as two significant three-way interactions between block, the social nature of the cues and the engaging nature of the cues (Wald 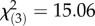, *p* = 0.002) and age, block and the social nature of the cues (Wald 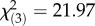, *p* < 0.001). Finally, a marginal four-way interaction between block, age, the social nature of the cues and the engaging nature of the cues (Wald 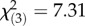, *p* = 0.063) was found. Planned follow-up contrasts revealed that, in the social condition, the groups of children made more rewarding choices when the cues were engaging compared to when they were non-engaging for the three last blocks 2, 3 and 4 (all *p* < 0.021) but not during the first block (*p* = 0.623) whereas the groups of adults made more rewarding choices when the cues were engaging compared to when they were non-engaging from the very first block (all *p* < 0.009). In the non-social condition, neither of the groups of children and adults showed differences in their choices towards the engaging and non-engaging cues in any of the blocks (all *p* > 0.098). No other interactions were significant.

## 4. Discussion

It is fundamental for social, cognitive and affective neuroscience to identify the mechanisms underlying the development of selective attention to social cues. To our knowledge, this study, using a novel gaze-contingent reward learning task, demonstrates for the first time that the nature of social signals affects how young children and adults learn cue–reward associations and adjust their visual attention towards relevant cues accordingly. The results show that both groups of children and adults learned to fixate more often on the cues associated with a subsequent reward delivery. Both groups of children and adults were able to learn the cue–reward association in the social and non-social condition for both types of engaging and non-engaging cues. The results clearly demonstrate that both young children and adults can learn, through their gaze behaviour, the association between a visual cue and a rewarding outcome. Critically, both groups of children and adults learned the cue-association more rapidly and more efficiently when the reward-predictive cues presented social and engaging signals than when the cues were social but non-engaging. Finally, the group of adults made more rewarding choices compared to the group of children, when the reward-predictive cues were social and engaging than social and non-engaging. Additionally, the group of adults did so from the very beginning of the task whereas the group of children showed differences in rewarding choices only from the second block. This is in line with our hypothesis predicting that adults would perform more efficiently and more rapidly than children, as they may be more experienced with cue–reward associations.

These findings suggest that the engaging nature of social cues convey information that influences both the speed and efficiency to learn cue–reward association and to adjust visual orienting. The capacity to use conspecifics' social signals should be adaptive for survival and acquisition of knowledge relevant to the social environment. Indeed, such a capacity can also be found in several non-human primate species. For example, capuchin monkeys have been shown to use conspecifics' emotional expressions towards objects to weigh whether those objects are worthy of selection or not [33]. Similarly, rhesus monkeys (raised in laboratories settings and not initially afraid of snakes) show fearful behaviour towards snake like toys after having repeatedly observed videos of peers showing fear towards snakes [34], demonstrating the importance of learning cue–outcome associations.

The gaze behaviour observed in the current study is more likely to reflect the learning of the association between the predictive cue and subsequent reward delivery, rather than the association between the identity of the cueing stimuli and their engaging or non-engaging reactions, for the following reasons. Firstly, participants preferentially fixated on the social non-engaging reward-predictive cue in the third block (children) and the fourth block of the task (both children and adults), indicating the occurrence of reward learning, but only later in the task. This indicates that, when the reward-predictive cue was social and non-engaging, both groups of children and adults learned to fixate on the stimulus associated with reward delivery. By contrast, if children and adults only preferred fixating the social engaging cue because of its intrinsic hedonic value, their accuracy would have decreased over time in the condition where the reward-predictive cue was non-engaging, which did not happen in the current study. Secondly, the magnitude of the adult participants' preference for social engaging and reward-predictive cues were not different from that of non-social reward-predictive cues. This is inconsistent with the prediction derived from the hedonic value hypothesis that participants should show stronger preference for social engaging cues than non-social cues.

Similarly, these results are unlikely to be due to an endogenous attentional shift in the direction of the cue's gaze or arrow [35], because the effect of engagement (i.e. pointing at versus away from the reward) was only present in the social condition, and not in the non-social condition in which arrows should have also cued attention [36]. In addition, a supplementary analysis of the viewing time of the cartoon reward for both social and non-social conditions and engaging and non-engaging cues showed no difference in effective exposure to the rewarding stimulus between conditions (see electronic supplementary material). This demonstrates that the non-engaging arrow did not prevent the learning of cue–reward association by generating an attentional shift away from the location of the reward delivery.

It is also unlikely that the current results can be fully explained by the difference in ‘complexity’ between social and non-social stimuli, which might hinder the deeper encoding and identification of non-social stimuli. Indeed, both groups of children and adults successfully learned the cue–reward association in both engaging and non-engaging non-social conditions, performing above chance level during the last three blocks of the non-social conditions. This finding clearly demonstrates that both groups of children and adults were able to encode, discriminate and differentiate between the two non-social stimuli, and acquire the preference for one (i.e. the rewarding cue) over the other stimulus. Additionally, both groups of children and adults made less rewarding choices when the cues were social and non-engaging compared to non-social and non-engaging. This is inconsistent with the claim that the differences in learning between the social and non-social cues could be fully attributed to the overall better encoding of faces due to increased complexity. Indeed, this hypothesis would predict a better learning with social cues than with non-social cues, a result not observed in the current study. Instead, this result could imply that social and non-engaging cues might slow down the reinforcement learning, which would merit further investigation.

A limitation of the current study, however, is the imperfect matching in sensory dimensions of the social and non-social stimuli. Despite the fact that the non-social stimuli were designed to minimize any features which might inadvertently elicit agency or animacy perception and generate social responses, they differ from social stimuli in several dimensions. Future studies are needed to identify the essential features in social and engaging stimuli that facilitate the learning of cue–reward associations. For example, one could use degraded or inverted images of faces to assess the key aspects of facial stimuli relevant to the current findings. Similarly, one could also ask whether the language used in the social engaging condition, compared to the non-linguistic vocal sound used in the social non-engaging condition is essential for the facilitation of learning observed in the social engaging stimuli.

This study provides unique insights regarding how children learn to attend to communicative social cues. Engaging social cues might inform the presence of rewarding events in immediate future, and allow more efficient attentional control and decision making. From when they are born [37], and as they develop until adulthood, children are (hopefully) more and more experienced with the association of communicative social signals and subsequent positive outcomes. As a result, attention to these predictive cues is more likely to lead to the acquisition of relevant knowledge and beneficial positive experience. Thus, the capacity to spontaneously gaze towards relevant social signals is advantageous for obtaining relevant information about rewarding outcomes during social interactions. Our current finding that attention is influenced by the rewards associated with positive social signals could help explain examples of adverse social learning sometimes observed in negative early family environments. For example, children from families with harsh parenting show atypical neural responses towards negative emotional stimuli [38]. These children have potentially experienced the association of negative stimuli with severe reactions within the familial setting. These findings suggest that associative learning may modulate social cue processing.

The present findings also give weight to the hypothesis that atypical social attention observed in children with autism and in infants at risk for autism [39,40] is linked to atypical reward processing [7,41], which might generate a barrier for social attention and social learning in this population. In this context, the use of social and interactive stimuli could be a crucial component of the current experimental design. Indeed, atypical gaze behaviour in individuals with autism is more prominent when they observe social-dynamic stimuli [42] or interactive stimuli [43] compared with static stimuli. More generally, it has been shown that participants who believed to be interacting with real people judged gaze direction differently from participants who did not, suggesting that interactivity between people influence the perception of social signals [44]. In addition to the interactive nature of the paradigm used in the current study, this novel task does not require verbal instruction, and allows for the assessment of social orienting and reward learning in young, non-verbal and/or atypical population such as infants and young children with autism.

## 5. Conclusion

To our knowledge, this study is the first to demonstrate that associated reward delivery triggers both young children and adults' visual attention towards social signals, while showing that reward learning is positively affected when the cues are socially engaging. Our findings suggest that social and engaging cues have positive incentive value, possibly because they usually co-occur with positive outcomes in everyday life, which could be a factor driving the development of social orienting.

## Supplementary Material

Supplementary information S2 (pdf): Additional analyses.

## Data accessibility

Supporting data are available on the Dryad Digital Repository: http://dx.doi.org/10.5061/dryad.s64sg [45].
