# Supplementary material for: Gaze-contingent reinforcement learning reveals incentive value of social signals in young children and adults
Source: Proc Biol Sci. 2017 Mar 1;284(1850):20162747. doi: 10.1098/rspb.2016.2747 (PMC5360925; doi:10.1098/rspb.2016.2747)
Supplement: Supplementary information S2 (pdf): Additional analyses. [file rspb20162747supp1.docx]

**Electronic supplementary material (ESM)**

1. **Exposure to both engaging and non-engaging cues.**
2. **Looking time for the reward-predictive cue at pre-test and post-test.**
3. **Side bias during pre-test and post-test.**
4. **Gaze-contingency awareness and reward appreciation.**
5. **Viewing time of the cartoon in all conditions.**
6. **Exposure to both engaging and non-engaging cues.**

Because of the gaze contingency nature of the task, the reactions of the cues can only be seen if the participants gazed at the cues. To ensure that the participants were exposed to the different reaction from the two cues at the very beginning of the task, the average number of trials reached before seeing either the reward-predictive or the penalty-predictive cue' reactions, was analysed for both adults and children groups in both social and non-social conditions and for both engaging and non-engaging cues. Wilcoxon Signed Rank Tests revealed that the average number of trials reached before seeing the reward-predictive cue (all Medians between 1 and 3 trials) was not significantly different from the average number of trials reached before seeing the penalty-predictive cue (all Medians between 1 and 2.5 trials), for both groups of children and adults, in both social and non-social conditions and for both type of engaging and non-engaging cues (all Z < 1.57, all p > .117). In addition, Mann–Whitney U tests revealed that the average number of trials reached before seeing the reward-predictive cues and the penalty-predictive cues did not differ between engaging and non-engaging cues for both groups of children and adults, in both social and non-social conditions (all U > 87.50, all p > .105). These results indicate that in all the conditions, both groups of children and adults were exposed to the reaction of both reward-predictive and penalty-predictive cues from the very beginning of the task. Therefore, the differences in rewarding first looks over the 40 trials reported in the main text, are unlikely to be due to a late exposure to the different reactions from the two cues.

1. **Looking time for the reward-predictive cue at pre-test and post-test.**

The proportion of looking time to the reward-predictive cue at pre-test and post-test, which were calculated by dividing the looking time towards the reward-predictive cue by the total looking time towards both the reward-predictive and penalty-related cues, was analysed in a 2 (test: pre-test, post-test) x 2 (social nature of the cues: social vs non-social) x 2 (engaging nature of the cues: engaging vs non-engaging) x 2 (age: children vs adults) mixed ANOVA. The analyses revealed a main effect of age (F (1,114) = 14.46, p < .001, η_p_^2^ = .11) and a significant main effect of test (F (1, 114) = 15.05, p < .001, η_p_^2^ = .12) that were qualified by a significant two-way interaction between test and age (F (1,114) = 23.03, p < .001, η_p_^2^ = .17). Follow up contrasts revealed that the group of adults looked significantly longer at the rewarding-predictive cue at post-test than at pre-test (t (58) = 5.95, p < .001, d = 1.10). This was not the case for the group of children (t (62) = .68, p = .503, d = .12). In addition, the analyses revealed a significant two-way interaction between test and the engaging nature of the cues (F (1,114) = 4.71, p = .032, η_p_^2^ = .04), and a marginal three-way interaction between test, the social nature of the cues and the engaging nature of the cues (F (1,114) = 3.87, p = .052, η_p_^2^ = .03). The follow up contrasts were conducted for each group of adults and children due to the lack of difference of looking time between pre-test and post-test observed in the group of children. Follow up contrasts revealed that the group of adults looked significantly longer at the rewarding-predictive cue at post-test than at pre-test, when the reward-predictive cue was social and engaging (t (14) = 4.74, p < .001, d = 1.73), non-social and engaging (t (13) = 3.87, p = .002, d = 1.46) and non-social and non-engaging (t (15) = 3.97, p = .001, d = 1.40), corroborating the higher number of rewarding first looks observed in the adults group over the course of the task, reported in the main text. There was, however, no differences in looking time towards the reward-predictive cue between pre-test and post-test when the reward-predictive cue was social and non-engaging (t (13) = .60, p = .557, d = .23) (Figure S1b and S1d). This lack of preferential looking towards the social and non-engaging reward predictive cue, is in line with the main analyses of rewarding first looks reported in the main text that showed slower learning of the cue-reward association when the reward-predictive cue was social and non-engaging in adults. The group of children did not show any preferential looking towards the reward-predictive cue for any of the conditions at pre, and post-test (all t < 1.68, all p > .114, (Figure S1a and S1c). This lack of preferential looking between pre-test and post-test observed in children may be due to a visual leftward bias [1]. A further analysis of a side bias during the preferential looking test at pre-test and post-test, was therefore conducted (section 3 in this document).

Figure S1. Proportion of looking time to the reward-predictive cue in both social a) and b) and non-social conditions c) and b) for both children a) and c) and adults b) and d). Proportion of looking time when the reward-predictive cue was engaging are in dark grey, Proportion of looking time when the reward-predictive cue was non-engaging are in light grey. (Errors bars: standard errors).

1. **Side bias during pre-test and post-test.**

The location of the reward-predictive cue, was either on the left or right of the screen at pre-test and post-test, and was counterbalanced across participants. However, it is possible that the lack of preferential looking towards the reward-predictive cue between pre-test and post-test observed in children may be masked by a visual leftward bias. To examine this hypothesis, looking time at pre-test and post-test was analysed with a 2 (test: 'pre-test', 'post-test') x 2 (side: left vs right) x 2 (age: children vs adults) mixed ANOVA. As well as corroborating the previously reported main effect of test (F (1,106) = 13.68, p < .001, η_p_^2^ = .11), main effect of age (F (1,106) = 16.71, p < .001, η_p_^2^ = .14), and interaction test and age (F (1,106) = 24.83, p < .001, η_p_^2^ = .19), the ANOVA revealed a main effect of side (F (1,106) = 16.71, p < .001, η_p_^2^ = .14). Follow up contrasts revealed no significant difference in looking time towards the reward-predictive cue at pre-test when the predictive cue was presented on either on the left or right side (M^left-side^ = .53, SD = .13; M^right-side^ = .50, SD = .18; t (121) = .99, p = .323, d = .18). However, the analyses revealed a significant difference in looking time towards the reward-predictive cue at post-test between the presentation of the predictive cue on the left side compared to the right side (M^left-side^ = .69, SD = .30; M^right-side^ = .55, SD = .32; t (121) = 2.45, p = .016, d = .44). In addition, despite the non-significant interaction between test, side and age (F(1,106) = 2.22, p = .139), explorative follow up contrasts were conducted for each group of adults and children to examine whether the lack of preferential looking towards the reward-predictive cue between pre-test and post-test observed in children may be masked by a visual leftward bias In adults, follow up contrasts revealed no significant differences in looking time between the presentation of the predictive cue on the left side compared to the right side at both pre-test (M^left-side^ = .53, SD = .14; M^right-side^ = .48, SD = .17; t(58) = 1.34, p = .185, d = .35) and post-test (M^left-side^ = .80, SD = .29; M^right-side^ = .72, SD = .30; t(58) = 1.08, p = .284, d = .28). However, in children, follow up contrasts revealed no significant differences in looking time between the presentation of the predictive cue on the left side compared to the right side at pre-test (M^left-side^ = .53, SD = .14; M^right-side^ = .52, SD = .18; t(61) = .07, p = .946, d = .02) but revealed a significant difference in looking time between left and right locations of the reward-predictive cue at post-test (M^left-side^ = .59, SD = .28; M^right-side^ = .39, SD = .27; t(61) = 2.80, p = .007, d = .71). These analyses reveal that rather than a looking time preference for the reward-predictive cue, a looking time preference for the left side is found at post-test for children. This measure seems to reflect an interesting bias but not an appropriate measure of learning in the group of children in this study.

1. **Gaze-contingency awareness and reward appreciation.**

Questions to the participating children:

**Awareness of the gaze contingency**: ‘The cartoon appeared on the screen several times, do you know what made the cartoon appear on the screen?’, ‘Did you see that if you looked at one of the ladies/balls, the cartoon appeared on the screen?’, ‘Which one of the ladies/balls, did you have to look at to make the cartoon appear on the screen?’

**Rewarding nature of the cartoon**: ‘At the moment, if at home you have the choice among these cartoons, which one do you prefer to watch?’ Five pictures of the most popular cartoons as of the years 2014/2015 (Peppa Pig, The Octonauts, Thomas & Friends, Bubble Guppies, Mike the Knight) were used to help answer this question. Every time a child chose his most favourite cartoon, the picture of the cartoon was removed and the question was repeated for the remaining cartoons. An order of preference of ‘Peppa pig’ among the other cartoons ranging from 1 to 5 was therefore obtained. All parents approved the order of preference obtained.

Questions to the participating adults:

**Awareness of the gaze contingency**: ‘Describe the task in a few sentences. What happened during the task?’, ‘Did you notice that you were in control of the task by looking at the ladies/buttons? (yes, no)’, ‘Which one of the ladies/buttons, did you have to look at to make the cartoon appear on the screen?’

**Rewarding nature of the cartoon**: ‘On a scale from 1 to 5 (1: ‘very unhappy’, 5: ‘very happy’): How did you feel when the cartoon appeared on the screen?’

Note: The experimenter questioned the children verbally and adult participants were given a questionnaire to fill. Adult participants answered questions 2, 3 and 4 on a different form after they had answered the first question to avoid to influence their initial feedback.

It is unclear whether children were aware of the gaze contingency, as most of them were not able to provide comprehensive answers to the questions related to their awareness of the rule of the task, possibly because of the complexity of the questions and/or shyness. However, 53 out of 64 adults reported to be aware of the gaze contingency. Participants’ feedback also indicated that most of children and adults appreciated watching the cartoon. Out of 62 children, 47 reported the cartoon employed for the experiment to be their favourite or second favourite among the other cartoons presented in the question (two children were not able to provide an order of preference of the cartoons). The numbers of children who reported the cartoon to be their third, fourth and last favourite cartoon were as follow: 6, 6 and 3. Out of 64, 58 adults scored 4 and 5 (5 being the maximum score on the happiness scale) to describe how happy they felt when the cartoon appeared on the screen. These descriptive statistics illustrate the positive response of both children and adults towards the cartoon reward used in this study. Interestingly, in the current study, 83% of the participating adults confirmed in a questionnaire their knowledge of gaze-contingency and use of the association between the reward and the non-social cues, contrasting with 36% of the adult participants who did so in a previous research [2]. The difference between the two studies might result from the type of non-social cues employed, which was static in Wang et al. [2] but dynamic in the current study. The movement of the arrow in the current study might have replicated the contingent response usually observed with social cues during social interactions, which could elicit social attention such as gaze following [3].

1. **Viewing time of the cartoon in all conditions.**

If the participants looked at the reward-predictive cue, then the cartoon was displayed for a maximum of six seconds. However, the duration of the presentation of the cartoon could be reduced if the participants looked at the penalty-predictive cue. To ensure that the participants were exposed to the cartoon for the same amount of time between conditions, the proportions of viewing time of the cartoon was computed (viewing time of the cartoon divided six) and analysed in a two-way ANOVA. The social nature of the cues (social/non-social) and the engaging nature of the cues (engaging/non-engaging) were entered in the ANOVA as between-subject factors. The analysis found no significant differences in looking time for the cartoon between social and non-social conditions (no main effect of condition, F (1,128) = 2.48, p = .118, η_p_^2^ = .02), between the engaging and non-engaging nature of reward-predictive cues (no main effect of engagement, F (1,128) = 1.98, p = .162, η_p_^2^ = .02) or the combination of these factors (no interaction between condition and engagement, F (1,128) = 1.53, p = .218, η_p_^2^ = .01). This analysis of the viewing time of the cartoon reward showed there is no difference in effective exposure to the rewarding stimulus (cartoon) in both social and non-social conditions and engaging and non-engaging cues. Therefore, the differences in rewarding first looks reported in the main text, are unlikely to be due to a reduced exposure of the reward.

**References**

[1] Marzoli D, Prete G, Tommasi L. Perceptual asymmetries and handedness : a neglected link ? 2014;5:1–9. doi:10.3389/fpsyg.2014.00163.

[2] Wang Q, Bolhuis J, Rothkopf CA, Kolling T, Knopf M, Triesch J. Infants in Control: Rapid Anticipation of Action Outcomes in a Gaze-Contingent Paradigm. PLoS One 2012;7:e30884. doi:10.1371/journal.pone.0030884.

[3] Deligianni F, Senju A, Gergely G, Csibra G. Automated gaze-contingent objects elicit orientation following in 8-month-old infants. Dev Psychol 2011;47:1499–503. doi:10.1037/a0025659.
